# Generation and characterization of an inducible renal proximal tubule-specific CreERT2 mouse

**DOI:** 10.3389/fcell.2023.1171637

**Published:** 2023-05-05

**Authors:** Shiting Liang, Youliang Wang, Meixia Kang, Juan Deng, Liting Chen, Xizhen Hong, Fan Fan Hou, Fujian Zhang

**Affiliations:** Division of Nephrology, Nanfang Hospital, Southern Medical University, National Clinical Research Center for Kidney Disease, State Key Laboratory of Organ Failure Research, Guangdong Provincial Institute of Nephrology, Guangdong Provincial Key Laboratory of Renal Failure Research, Guangzhou, China

**Keywords:** AMN, AMN-CreERT2, renal proximal tubule, protein reabsorption, CRISPR/Cas9

## Abstract

Protein reabsorption in renal proximal tubules is essential for maintaining nutrient homeostasis. Renal proximal tubule-specific gene knockout is a powerful method to assess the function of genes involved in renal proximal tubule protein reabsorption. However, the lack of inducible renal proximal tubule-specific Cre recombinase-expressing mouse strains hinders the study of gene function in renal proximal tubules. To facilitate the functional study of genes in renal proximal tubules, we developed an *AMN* ^*CreERT2*^ knock-in mouse strain expressing a Cre recombinase–estrogen receptor fusion protein under the control of the promoter of the *amnionless (AMN)* gene, a protein reabsorption receptor in renal proximal tubules. *AMN* ^*CreERT2*^ knock-in mice were generated using the CRISPR/Cas9 strategy, and the tissue specificity of Cre activity was investigated using the Cre/loxP reporter system. We showed that the expression pattern of CreERT2-mEGFP in *AMN* ^*CreERT2*^ mice was consistent with that of the endogenous *AMN* gene. Furthermore, we showed that the Cre activity in *AMN* ^*CreERT2*^ knock-in mice was only detected in renal proximal tubules with high tamoxifen induction efficiency. As a proof-of-principle study, we demonstrated that renal proximal tubule-specific knockout of *Exoc4* using AMN^CreERT2^ led to albumin accumulation in renal proximal tubular epithelial cells. The *AMN* ^*CreERT2*^ mouse is a powerful tool for conditional gene knockout in renal proximal tubules and should offer useful insight into the physiological function of genes expressed in renal proximal tubules.

## Introduction

The kidney is a crucial organ responsible for blood filtration, protein reabsorption, hormone secretion, and the removal of endogenous waste products. Over the past decade, numerous genes associated with kidney diseases have been identified through whole-exome sequencing or targeted gene sequencing. Despite these progresses, the underlying molecular mechanisms remain to be fully elucidated (Kumar, 2018). Gene knockout animals obtained through gene targeting have been essential tools in addressing this question (Paquet et al., 2016). However, the traditional knockout technique has inherent limitations. For instance, global knockout of a gene with a significant function can be embryonic lethal in homozygous animals, precluding analysis of the phenotype in adult knockout animals (Starremans et al., 2008; Wu et al., 2017). To address these limitations and assess gene function in adults, the tissue-specific gene knockout approach has been developed. This approach involves two mouse lines, one containing a floxed target gene and the second expressing the Cre recombinase under the control of a tissue- or cell-specific promoter. The Cre recombinase mediates the recombination between two loxP sites, resulting in gene insertion or deletion. Thus, tissue or cell specificity of conditional gene knockout is determined by the expression of the Cre recombinase in a specific tissue or cell type (Sternberg, 1981; Van Duyne, 2015).

Renal proximal tubules reabsorb proteins that pass through the glomerular filtration barrier. Defects in renal proximal tubule reabsorption lead to renal Fanconi syndrome, characterized by low-molecular-weight tubular proteinuria, glycosuria, phosphaturia, aminoaciduria, bicarbonaturia, and uricosuria (Tolaymat et al., 1992; Klootwijk et al., 2014). Renal proximal tubules are highly vulnerable to various injuries, such as ischemic reperfusion injury, hypoxia, and toxins (Liu et al., 2018). Thus, the study of the function of genes responsible for protein reabsorption in renal proximal tubules is essential for elucidating the molecular mechanisms underlying various renal tubular diseases. Currently, several transgenic mouse strains that express Cre recombinase in renal proximal tubules have been reported, including those controlled by the promoters of kidney androgen-regulated protein (*KAP*), gamma–glutamyl transpeptidase (*γGT*), sodium–glucose co-transporter type 2 (*SGLT2*), and phosphoenolpyruvate carboxykinase (*PEPCK*). However, *PEPCK-Cre* (Rankin et al., 2006), *SGLT2-Cre* (Rubera et al., 2004), and *γGT-Cre* (Iwano et al., 2002) mouse strains constitutively expressing Cre recombinase are not suitable for tissue or cell-specific gene inactivation at the adult stage. In contrast, the *KAP2-Cre* mouse line has the advantage of being highly tissue specific, and its activity can be regulated by exogenous androgen administration in female mice (Li et al., 2008). Moreover, a *GGT-CreERT2* mouse line was developed, in which the CreERT2 fusion protein is driven by the mouse γ-glutamyl transpeptidase type II gene promoter, and its expression is only detected in the S3 segments of the proximal tubules (Dworniczak et al., 2007). In the *SLC34a1-CreERT2* strain, CreERT2 is expressed in the S1, S2, and cortical S3 segments, but not in the medullary S3 segment (Kusaba et al., 2014).

Cubilin and AMN are two major receptors responsible for receptor-mediated vitamin B12 uptake in the small intestine and protein reabsorption in renal proximal tubules (Birn et al., 2000). Both are specifically expressed in the small intestine epithelium and renal proximal tubules. The exocyst is a highly conserved octameric protein complex that mediates the tethering of secretory vesicles or recycling endosomes before membrane fusion (He and Guo, 2009). Mutations in exocyst genes have been identified in patients with kidney disease (Nihalani et al., 2019). A previous study has also shown that exocyst genes are essential for protein reabsorption in *Drosophila* nephrocytes (Wen et al., 2020). Thus, we reasoned that it is suitable to generate an inducible proximal tubule-specific CreERT2 line under the control of the endogenous *AMN* gene promoter to study the function of genes involved in renal proximal tubular protein reabsorption, such as exocyst genes.

In this study, we report the generation and characterization of a knock-in mouse strain expressing a tamoxifen-inducible Cre recombinase, specifically in the entire renal proximal tubule. A Cre-ERT2-P2A-mEGFP cassette was inserted before the stop codon of the mouse *AMN* gene *via* homologous recombination using the CRISPR/Cas9 system. We demonstrated that this AMN^CreERT2^ induces cell-specific recombination in renal proximal tubules with high induction efficiency. As a proof-of-concept study, renal proximal tubular knockout of the *Exoc4* gene using *AMN* ^*CreERT2*^ led to protein degradation defects in renal proximal tubular cells. Our results illustrate that this new *AMN* ^*CreERT2*^ mouse allows tissue-specific knockout of genes in renal proximal tubules and can facilitate the study of the effect of these genes on renal proximal tubular protein reabsorption.

## Materials and methods

### Mice

The mice were maintained in specific pathogen-free conditions in the Laboratory Animal Resource Center at Nanfang Hospital. They were kept in a room with a temperature-controlled environment (23°C–25°C), a 12:12-h light–dark cycle and had unlimited access to food and water. The *AMN-CreERT2-mEGFP* knock-in mice strain was generated by Biocytogen Pharmaceuticals (Beijing) Co., Ltd., and *C57BL/6* mice were used in the maintenance process. The *Rosa26-loxP-Stop-loxP-tdTomato* (*Rosa26-LSL-tdTomato*) mice were provided by Professor Bin Zhou and used for the analysis of Cre recombination activity. The *Exoc4* ^*fl/fl*^ mice were provided by GemPharmatech. All animal studies were carried out following the Guide for the Care and Use of Laboratory Animals and were approved by the Experimental Animal Committee at the Nanfang Hospital, Southern Medical University.

### Generation of *AMN-CreERT2-mEGFP* knock-in mice

The *AMN-CreERT2-mEGFP* knock-in mice (*AMN* ^*CreERT2*^ mice) were generated *via* the CRISPR/Cas9 approach on the *C57BL/6N* background. Two sgRNAs were designed using the CRISPR design tool (http://www.sanger.ac.uk/) to target the region between exon12 and 3′UTR and were then screened for on-target activity using the Universal CRISPR Activity Assay (UCATM, Biocytogen Pharmaceuticals Co., Ltd.). SgRNA1 (GAG​AGA​AGT​CAC​GGT​CTC​CGA​GG) and sgRNA2 (CAG​AGG​CCT​GAC​CTG​CTA​GAA​GG) were selected for *in vitro* transcription to obtain the gRNA targeting the insertion site. The gene targeting vector containing a 5′homologous arm, P2A-CreERT2-P2A-mEGFP cassette, and 3′homologous arm was used as a template to repair the DSBs generated by Cas9/sgRNA. The P2A-CreERT2-P2A-mEGFP cassette was inserted before the stop codon of the mouse *AMN* gene using homologous recombination. A T7 promoter sequence was added to the Cas9 and sgRNA template by PCR amplification for *in vitro* transcription. Cas9 mRNA, targeting vector, and sgRNAs were co-injected into the cytoplasm of one-cell stage fertilized *C57BL/6N* mouse eggs. F0 mice with the expected genotype were confirmed by tail genomic DNA PCR and sequencing, and they were mated with *C57BL/6N* mice to establish germline-transmitted F1 heterozygous mice. F1 heterozygous mice were genotyped by tail genomic DNA PCR, southern blot, and DNA sequencing. The 5′set of primers (Forward1 CTC​TTC​CCG​CGC​GAT​GGA​TCT​TTC​C and Reverse1 TCATGC GGA​ACC​GAG​ATG​ATG​TAG​C) generates a 3.2 kb PCR product for the mutant allele. The 3′set of primers (Forward2 GCCAGGCTTTGT GGATTTGACCCTCC and Reverse2 TTC​CCT​ACC​CAG​GGT​GCA​ATT​TGT​T) generates a 4.1 kb PCR product for the mutant allele. The restriction enzymes Xmn I and Stu I were used to digest the genomic DNA for Southern blot analysis. The 5′probe was used to detect whether the donor vector was correctly inserted in the desired site. If the correct recombination occurred, two DNA bands (3.8 and 3.2 kb) would appear. The EGFP probe was used to examine whether there was off-target insertion. If there were no off-target insertions, only one on-target DNA band (4.1 kb) would appear.

### Mice genotyping

Genomic DNA was prepared from the mouse tail. Tissues were lysed by incubation with proteinase K overnight at 55°C, followed by centrifugation at 12,000 g for 10 min to obtain supernatant with genomic DNA. Specific primers were used to distinguish the mutant allele from the wild-type allele. The genotyping primers are as follows: AMN-CreERT2 (mutant: 347 bp, WT: negative).

Forward: 5′- CAG​CAG​GTG​AGA​CTG​TGG​GAA​GAA​G-3′.

Reverse: 5′- CCT​GAC​TTC​ATC​AGA​GGT​GGC​ATC​C-3′.

### Tamoxifen injection

To induce Cre recombination in *AMN* ^*CreERT2*^ *; Rosa26-LSL-tdTomato* mice, tamoxifen (T832955, Macklin) was dissolved in a 1:4 ratio of ethanol and corn oil. The injection was given intraperitoneally to eight-week-old mice. The control group received injections of the vehicle (ethanol and corn oil) on day 1 and day 3. The low-dose group received 100 mg/kg tamoxifen on day 1 and day 3. The medium-dose group received tamoxifen injection (100mg/kg) every other day for 10 days. The high-dose group received tamoxifen injection (150 mg/kg) every other day for 10 days. The aforementioned groups of animals were euthanized on day 14 after the first administration of tamoxifen for further analysis.

The *AMN* ^*CreERT2*^ *; Exoc4* ^*fl/fl*^ mice were eight weeks old and divided into two groups for the study. The tamoxifen-treated group received tamoxifen injections (100 mg/kg) every other day for 10 days. The vehicle-treated group was injected with corn oil. After the first administration, the animals were euthanized on day 14 for quantitative real-time PCR analysis and immunostaining.

### Tissue preparation and histology

In brief, mice were anesthetized, euthanized, and perfused through the left ventricle with ice-cold PBS. Kidneys were hemi-sectioned, and portions were snap-frozen in liquid nitrogen. Other parts of the kidneys were fixed in 4% neutral buffered formalin at 4°C for 12 h, processed, and embedded in paraffin wax for subsequent analysis. Some kidneys were fixed in 4% PLP fixative (4% paraformaldehyde, 75 mM L-lysine, and 10 mM sodium periodate) for 2 h at 4°C, incubated in 30% sucrose, and snap-frozen in O.C.T Compound (Tissue-Tek, Sakura Finetek). Cryosections were mounted on Fisher Superfrost Plus microscope slides for immunofluorescence staining, as described in the following section.

### Immunofluorescence staining

All tissues were collected and fixed in 4% PLP fixative at room temperature for 1 h. The samples were then incubated in 30% sucrose/1x phosphate-buffered saline (PBS) overnight at 4°C, embedded in O.C.T Compound (Tissue-Tek, Sakura Finetek), and quick-frozen at −80°C until use. Then, 3 µm-thick tissue sections were transversely cut through the entire kidney using a cryotome (Leica Microsystems, Germany) and mounted on microscope slides for immunofluorescence staining. The 3 µm slides were thawed for 30 min at 4°C and washed with PBST (PBS, 0.3% Triton X-100). After blocking with 5% bovine serum albumin in PBST at room temperature for 1 h, the slides were immunostained with primary antibodies against Aqp1 (20333-1-AP, Proteintech, 1:100), NKCC2 (18970-1-AP, Proteintech, 1:100), megalin (sc-515772, Santa Cruz, 1:100), Calbindin (66,394-1, Proteintech, 1:50), Aqp2 (ab62628, Abcam, 1:100), podocalyxin (AF1556, R&D Systems, 1:100), and GFP (50,430-2, Proteintech, 1:100) at 4°C overnight. The slides were then washed in PBST and PBS successively and incubated in secondary antibody solution at room temperature for 2 h. The secondary antibodies were diluted in PBST. The following secondary antibodies were used: Alexa Fluor^®^ 488 Donkey Anti-Goat IgG (ab150129, Abcam, 1:400), Cy3-AffiniPure Donkey Anti-Rabbit IgG (715-165-150, Jackson ImmunoResearch, 1:200), Cy2-AffiniPure Donkey Anti-Rabbit IgG (111-225-144, Jackson ImmunoResearch, 1:200), and Cy2-AffiniPure Donkey Anti-Mouse IgG (715-225-150, Jackson ImmunoResearch, 1:200). After washing, nuclei were stained with DAPI (diluted 1:100,000 in PBS, Beyotime) at room temperature for 5 min. The slides were viewed under an Olympus BX61 microscope with all parameters held constant throughout.

### Immunohistochemistry

The renal tissues were fixed in 4% PLP fixative for 24 h and embedded in paraffin. Then, 2 μm-thick tissue sections were transversely cut through the entire kidney using a rotary microtome (Leica Microsystems, Germany) and mounted on microscope slides for immunohistochemistry staining. Antigens were retrieved by the microwave antigen retrieval method using citric acid buffer (pH 6.0). The slides were then incubated in methanol/hydrogen peroxide. After blocking with 5% bovine serum albumin in TBST at room temperature for 1 h, 2 μm tissue sections were incubated with megalin (sc-515772, Santa Cruz, 1:100), amnionless (sc-365384, Santa Cruz, 1:50), and albumin (16475-1-AP, Proteintech) antibodies overnight at 4°C, followed by incubation with the Biotin-SP AffiniPure Goat Anti-Mouse (Yeasen, 33203ES60,1: 400) secondary antibody for 1 h. After washing, the slides were incubated with streptavidin solution (Yeasen, 35105ES60, 1:300) for 30 min. AEC working solution (DAKO, K346111-2) was applied to cover the tissue section completely, and color development was monitored at room temperature using an Olympus DP27 microscope. The reaction can be terminated with pure water, and nuclei were counterstained with hematoxylin. Color images were acquired using the Olympus DP27 microscope.

### Quantification of tamoxifen induction efficiency and AMN^CreERT2^ specificity in renal proximal tubules

Kidney sections from *AMN* ^*CreERT2*^ *; Rosa26-LSL-tdTomato* mice were immunofluorescently stained with Aqp1 antibody to label the renal proximal tubules, as described earlier. Five images were randomly selected from each mouse kidney tissue (*n* = 4), and the numbers of Aqp1^+^ and Aqp1^+^/tdTomato^+^ cells were counted. Tamoxifen induction efficiency was defined as the ratio of Aqp1^+^/tdTomato^+^ cells to Aqp1^+^ cells, while the specificity was defined as the ratio of tdTomato^+^/Aqp1^+^ cells to tdTomota^+^ cells.

### Reverse transcription PCR

Total RNA was isolated using RNAiso Plus (TAKARA), and 1 μg of RNA was reverse transcribed using the PrimeScript™RT Reagent Kit with gDNA Eraser (Takara). Specific primers were used to amplify *Exoc4* mRNA and distinguish the truncated product from the wild-type form. The primers are as follows:

Forward primer: CTG​CTC​ATC​TCG​GTG​ATC​AG.

Reverse primer: TGA​GGT​TTT​GGT​GAC​GCC​TT.

### Quantitative real-time PCR

Reverse transcription PCR was performed as described previously. Quantitative real-time PCR was conducted on an ABI PRISM 7000 sequence detection system (Thermo Fisher Scientific, Waltham, MA) using SYBR Green Master Mix. The primer sequences for the *Exoc4* and *β-actin* genes are as follows:

*Exoc4* gene:

Forward primer: GCC​AGC​AAG​CAC​TAC​CTC​AG.

Reverse primer: CTT​GTT​ACG​CTG​TAC​CAC​CA.

*β-Actin* gene:

Forward primer: GAG​CGC​AAG​TAC​TCT​GTG​TG.

Reverse primer: AAC​GCA​GCT​CAG​TAA​CAG​TC.

### Urinary protein analysis

Following the initial administration of tamoxifen, the mice were housed in metabolic cages to collect 24-h urine samples on week 2 and week 8. Protein concentration was determined using the BCA Protein Assay Kit (Bio Vision), following the manufacturer’s instructions. The 24-h urine protein was calculated as the protein concentration (mg/mL) multiplied by the urine volume (mL). Spot urine samples free of fecal contamination were collected and subjected to SDS-PAGE electrophoresis. SDS-PAGE gels were then stained with Coomassie Brilliant Blue R250 (Sigma), and images were acquired using the Olympus SZX10 stereo microscope.

### Quantification and statistical analysis

Image quantification was performed using ImageJ, and statistical analysis was performed using SPSS version 24.0 (SPSS Inc., Chicago, IL, United States) and GraphPad Prism version 8.0 software (GraphPad Software, La Jolla, CA, United States). Student t-tests were used for comparing two groups, and a *p*-value of less than 0.05 was considered statistically significant.

## Results

### Generation of *AMN* ^*CreERT2-P2A-mEGFP*^ knock-in mice using the CRISPR/Cas9 strategy

**FIGURE 1:**
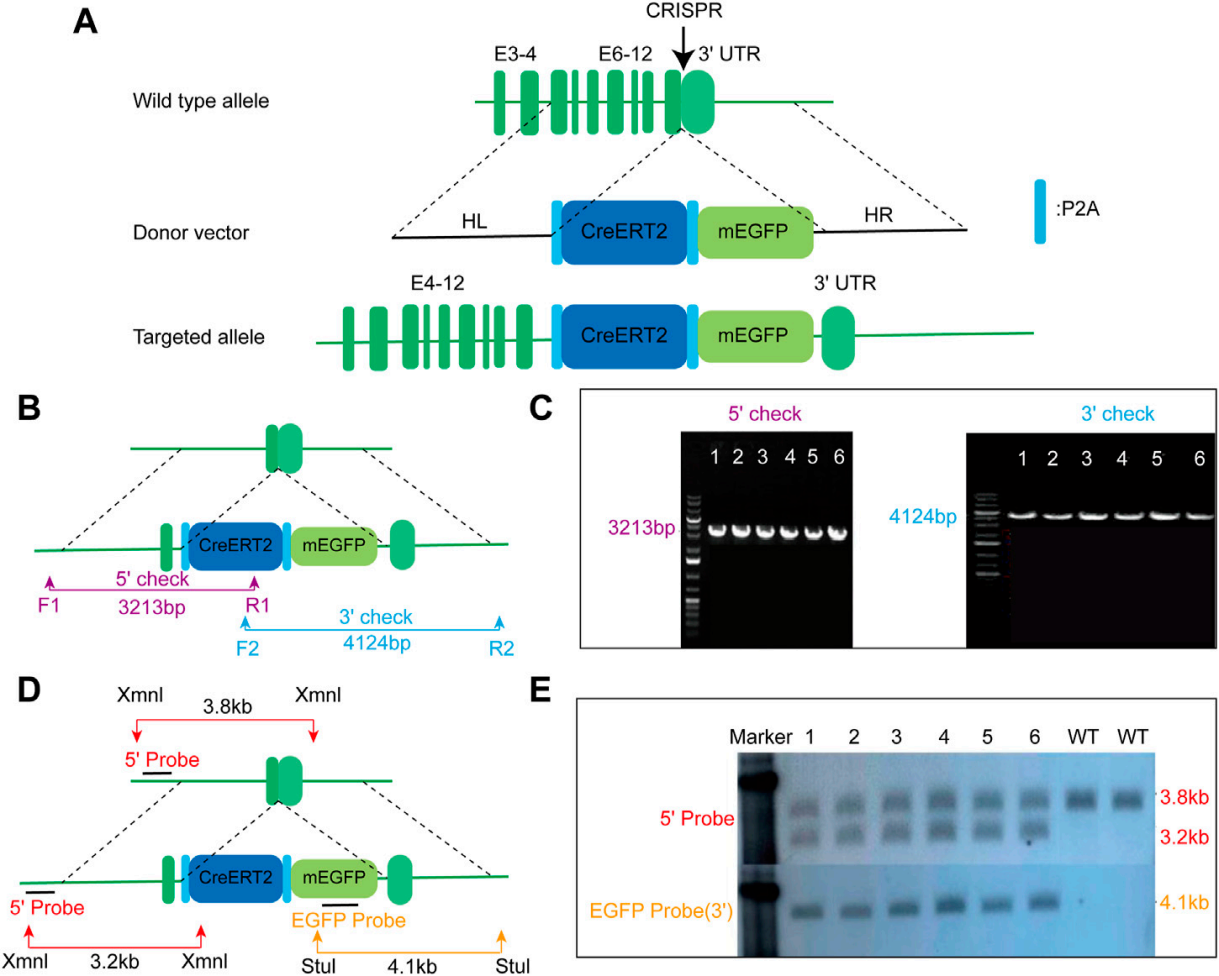
Generation of *AMN* ^*CreERT2-mEGFP*^ knock-in mice using the CRISPR/Cas9 strategy. **(A)** Schematic drawing depicting the wild-type allele of the mouse *AMN* gene, donor vector, and targeted allele. The donor construct, containing 1.5 kb 5′homologous arm (HL), 1.4 kb 3′homologous arm (HR), and the P2A-CreERT2-P2A-mEGFP gene cassette, was inserted into the last exon of the mouse *AMN* gene prior to the stop codon. E denotes exon. **(B)** Schematic diagram depicting the strategy for verifying on-target insertion using genomic PCR. The 5′set (purple) and 3′set (blue) of primers were used to confirm the precise insertion of the P2A-CreERT2-mEGFP-P2A cassette. **(C)** Representative agarose gel image of the PCR product from positive clones. The mutant allele for the 5′check was 3213 bp, while the mutant allele for the 3′check was 4124 bp. **(D)** Schematic representation depicting the strategy for confirming the mice with correct insertion using Southern blot. Xmn I (red arrow) and Stu I (yellow arrow) were utilized to digest the genomic DNA for Southern blot analysis. The 5′probe and EGFP probe were used to confirm accurate insertion of the P2A-CreERT2-mEGFP-P2A gene cassette. **(E)** Representative Southern blot image of positive clones using two different probes. The upper panel displays the blot result using the 5′probe, while the lower panel represents the result using the EGFP probe.

To drive the expression of CreERT2 fusion protein under the control of the mouse endogenous *AMN* gene, the P2A-CreERT2-P2A-mEGFP gene cassette was precisely inserted before the stop codon of the mouse *AMN* gene using the CRISPR/cas9 method (Figure 1A). F0 mice with the expected genotype were mated with *C57BL/6N* mice to establish germline-transmitted F1 heterozygous mice. F1 heterozygous mice were genotyped by tail genomic DNA amplification, Southern blot, and DNA sequencing. The result of genomic DNA amplification confirmed the insertion of the CreERT2-P2A-mEGFP gene cassette in six out of 22 pups (Figures 1B, C). Southern blot analysis also confirmed the correct insertion of the CreERT2-P2A-mEGFP gene cassette with no random insertions (Figures 1D, E). This mouse line was named *AMN* ^*CreERT2*^.

### Renal proximal tubular epithelial cell-specific expression of the inducible AMN^CreERT2^

**FIGURE 2:**
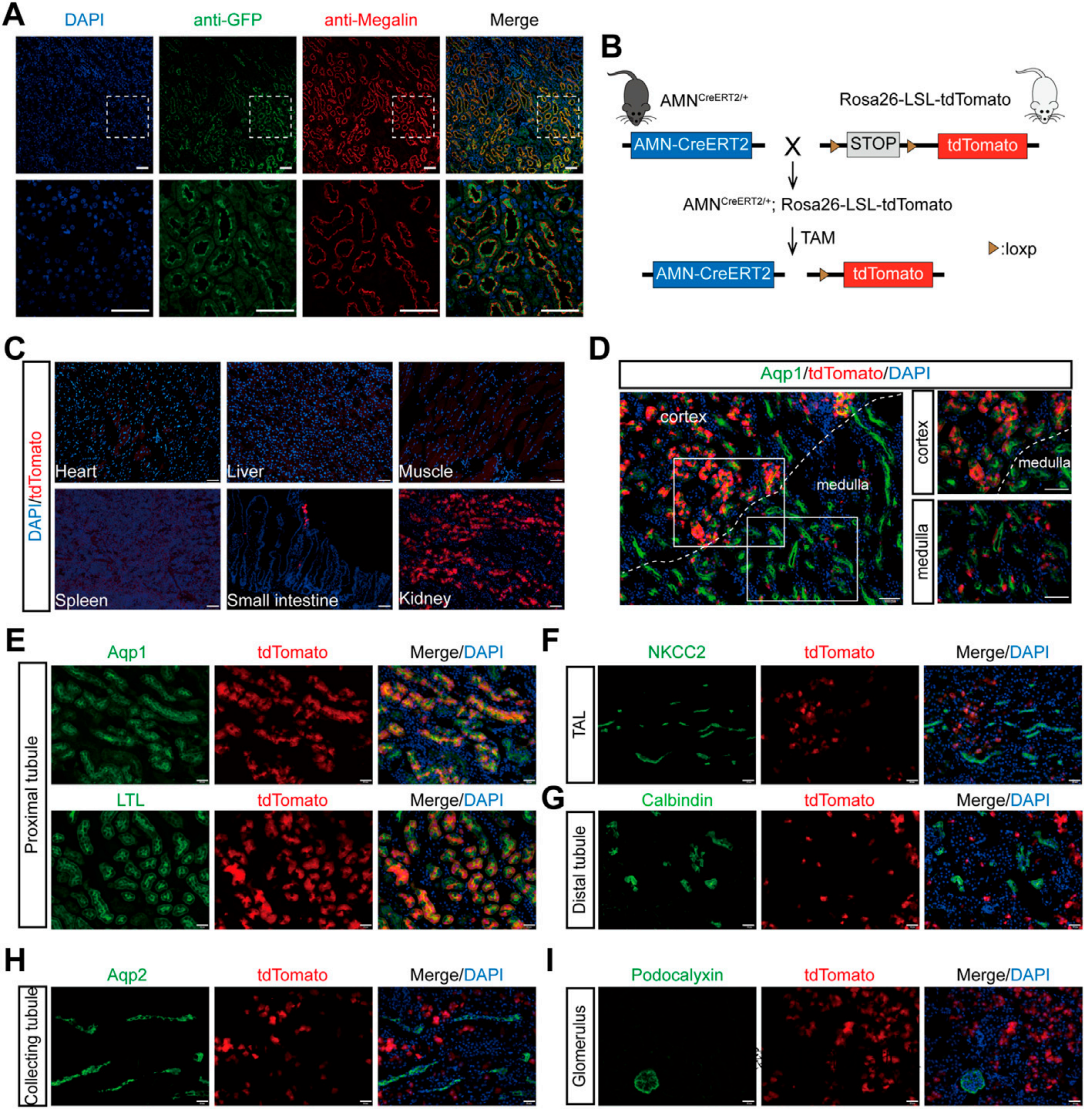
Renal proximal tubular epithelial cell-specific expression and activation of the Cre recombinase in *AMN* ^*CreERT2*^ mice. **(A)** Representative images showing the co-localization of GFP (green) and megalin (red) in renal proximal tubules. Rectangles depicted in the upper column were shown at a higher magnification in the lower column. **(B)** Diagram illustrating the generation of *AMN* ^*CreERT2*^ *; Rosa26-LSL-tdTomato* mice. **(C)** Expression of the tdTomato reporter gene in various tissues. The Cre activity (tdTomato^+^) was only detected in the small intestine and kidney of the *AMN* ^*CreERT2*^ *; Rosa26-tdTomato* mice following tamoxifen induction. **(D–I)** The Cre activity was detected in renal proximal tubules in the cortex and medulla, which was co-stained with Aqp1 and LTL **(D–E)**. No Cre activity was detected in the thick ascending limb of the loop of Henle stained with NKCC2 **(F)**, distal tubules stained with calbindin **(G)**, collecting tubules stained with Aqp2 **(H)**, and the glomerulus stained with podocalyxin **(I)**. Scale bar = 100 μm **(C, D)** and 50 μm **(A, E, F, G, H, I)**. The high dose of tamoxifen was used in this set of experiments.

To evaluate whether the Cre expression pattern in *AMN* ^*CreERT2*^ mice is consistent with that of the endogenous *AMN* gene, we performed immunofluorescent staining with an anti-GFP antibody and examined the co-localization of membrane EGFP and megalin, the renal proximal tubule marker. As shown in Figure 2A, the amplified mEGFP signal was completely co-localized with megalin. We also demonstrated that AMN is co-localized with megalin in renal proximal tubules by immunohistochemistry staining of serial sections (Supplementary Figure S1). These results showed that the Cre expression in *AMN* ^*CreERT2*^ mice is consistent with the expression pattern of the endogenous *AMN* gene.

To evaluate the tamoxifen-induced Cre activity of *AMN* ^*CreERT2*^ mice in different tissues and cells, the *AMN* ^*CreERT2*^ mice were mated with *Rosa26-LSL-tdTomato* reporter mice to generate *AMN* ^*CreERT2*^ *; Rosa26-LSL-tdTomato* mice. These mice express tdTomato red fluorescent protein after the removal of the stop cassette mediated by Cre recombinase (Figure 2B). Without tamoxifen induction, there were no tdTomato signals detected in any organs of *AMN* ^*CreERT2*^ *; Rosa26-LSL-tdTomato* mice. With tamoxifen injection, the tdTomato signal was only detected in the kidney and the small intestine, while no tdTomato signals were detected in the heart, liver, spleen, and muscle tissues (Figure 2C). In the kidney, the tdTomato-positive cells were mainly localized in the cortex and corticomedullary junction (Figure 2D). To further evaluate the cell specificity of the Cre recombination activity in the kidney, we performed immunofluorescent staining using different tubular-specific markers. As shown in Figure 2E, all the tdTomato-positive cells were co-stained with LTL and Aqp1, but not with the signals of NKCC2 (Figure 2F), calbindin (Figure 2G), Aqp2 (Figure 2H), and podocalyxin (Figure 2I). These findings suggest that tamoxifen-induced Cre activity in *AMN* ^*CreERT2*^ mice is specifically restricted to renal proximal tubular cells.

**FIGURE 3:**
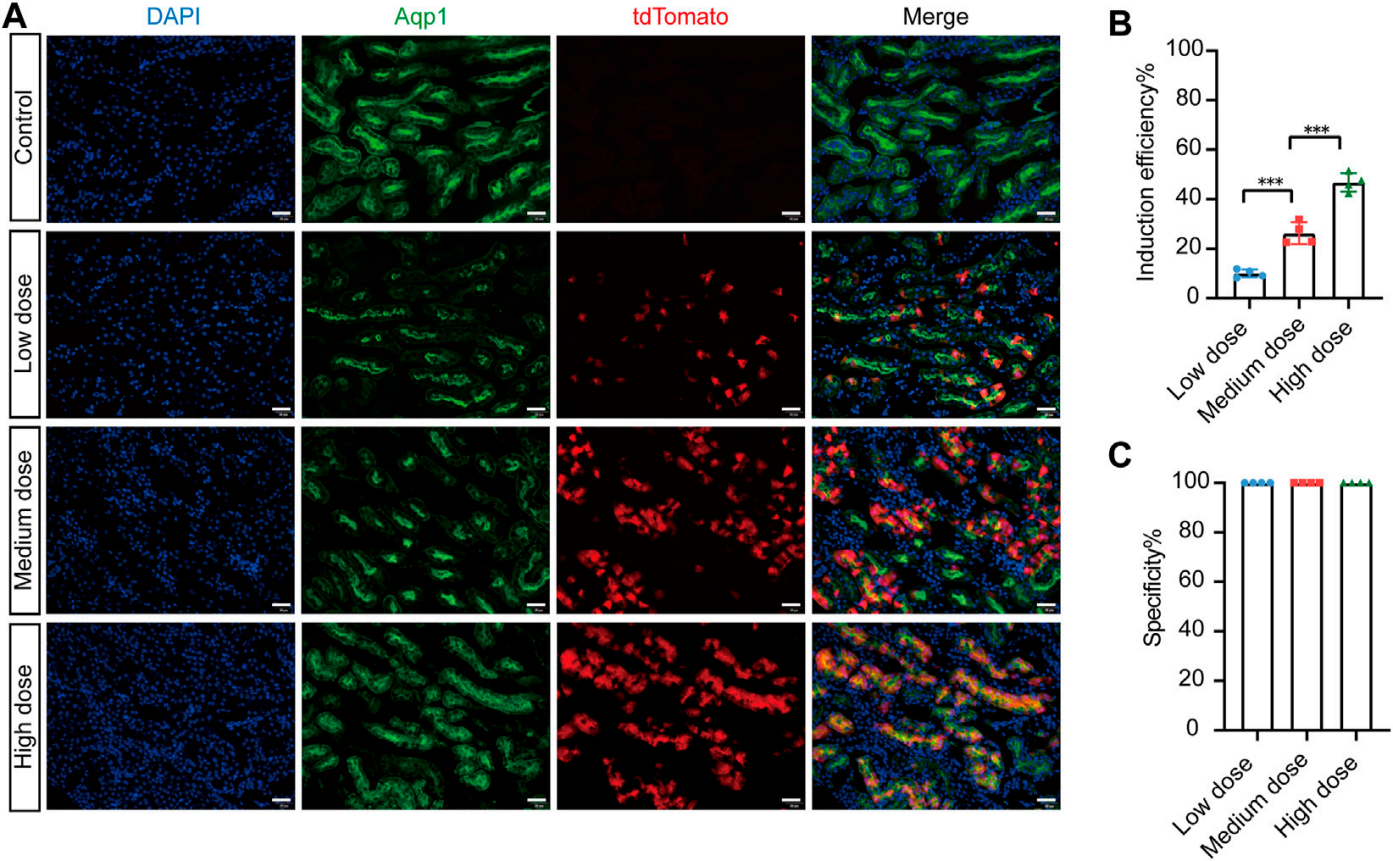
Efficient recombination occurred in renal proximal tubular cells of *AMN* ^*CreERT2*^ *; Rosa26-LTL-tdTomato* mice after tamoxifen administration. **(A)** The induced Cre recombination efficiency in proximal tubule epithelial cells of *AMN* ^*CreERT2*^ mice with different dosages of tamoxifen. In the control group, no leak Cre activity was observed in the whole kidney without tamoxifen induction. The total number of tdTomato-positive cells significantly increased with the rise in tamoxifen dosage. **(B)** Induction efficiency in different tamoxifen dose groups. The ratio of Aqp1^+^/tdTomato^+^ cells to Aqp1^+^ cells was measured in five randomly selected images per kidney (*n* = 4). Three asterisks signify highly significant differences between different groups. (**p < 0.001). **(C)** Cell specificity of CreERT2 activity in the renal proximal tubular epithelial cells. The ratio of Aqp1^+^/tdTomato^+^ cells to Aqp1^-^/tdTomato^+^ cells in five randomly selected images were measured per kidney (*n* = 4). Scale bar = 50 μm.

To investigate the tamoxifen induction efficiency of Cre-mediated recombination, we injected different doses of tamoxifen into *AMN* ^*CreERT2*^ *; Rosa26-LSL-tdTomato* mice and evaluated the induction efficiency. In the control group, there was no leaky Cre activity in the entire kidney without tamoxifen induction (Figure 3A). With increasing tamoxifen dosage, the number of tdTomato-positive cells increased significantly (Figure 3A). Our results showed that the tamoxifen induction efficiency was 10.09% ± 0.82% in the low-dose group, 26.30% ± 2.21% in the medium-dose group, and 46.80% ± 1.88% in the high-dose group (Figure 3B). The specificity of Cre activity in renal proximal tubular epithelial cells was 100% in all groups (Figure 3C). In summary, these data suggest that the *AMN* ^*CreERT2*^ mouse has completely restricted Cre recombination activity in renal proximal tubular epithelial cells, with very high tamoxifen induction efficiency.

### Tissue-specific knockout of the *Exoc4* gene in renal proximal tubules leads to protein degradation defect

**FIGURE 4:**
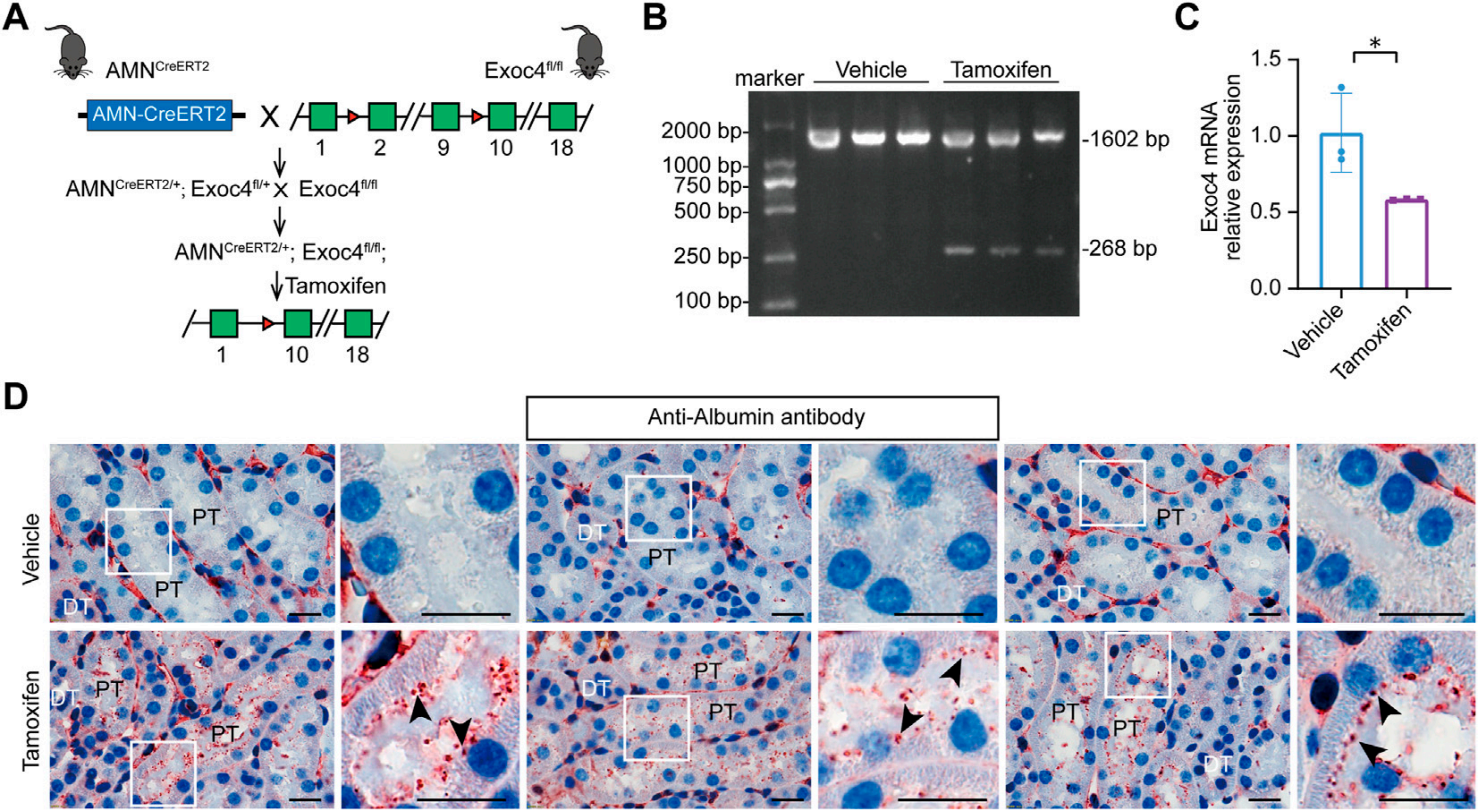
Knockout of the *Exoc4* gene, specifically in renal proximal tubules in *AMN* ^*CreERT2*^ *; Exoc4* ^*fl/fl*^ mice, led to albumin accumulation in the proximal tubular epithelial cells. **(A)** Diagram depicting the generation of tamoxifen-inducible *AMN* ^*CreERT2/+*^ *; Exoc4* ^*fl/fl*^ mice. **(B)** Representative agarose gel image of RT-PCR products from renal cortices of vehicle- or tamoxifen-treated *AMN* ^*CreERT2/+*^ *; Exoc4* ^*fl/fl*^ mice. The wild-type form of *Exoc4* mRNA amplification product is 1602 bp, while the truncated form is 268 bp. **(C)** mRNA expression levels of *Exoc4* in *AMN* ^*CreERT2/+*^ *; Exoc4* ^*fl/fl*^ mice at day 14 after vehicle (corn oil) or tamoxifen administration using RT-qPCR (*n* = 3). An asterisk denotes a significant difference between two groups (*p < 0.05). **(D)** Immunohistochemistry staining of albumin accumulation in renal proximal tubules of *AMN* ^*CreERT2/+*^ *; Exoc4* ^*fl/fl*^ mice at day 14 after vehicle or tamoxifen administration (*n* = 3). Rectangles depicted in the left column were shown at a higher magnification in the right column, respectively. The black arrowhead indicates albumin accumulation in the brush border of proximal tubular epithelial cells. PT denotes proximal tubule; DT denotes distal tubule. Scale bar = 20 μm.

The exocyst complex plays an important role in protein reabsorption in *Drosophila* nephrocytes. To evaluate whether the *AMN* ^*CreERT2*^ strain can be used to delete *Exoc4* in renal proximal tubules and investigate its effect on protein reabsorption, we generated *AMN* ^*CreERT2*^ *; Exoc4* ^*fl/fl*^ mice (Figure 4A). Our RT-PCR result showed that the expected recombination did happen, and a truncated form of *Exoc4* mRNA was present after tamoxifen induction but not present before the induction (Figure 4B). To evaluate the gene knockout efficiency of this *AMN* ^*CreERT2*^ strain, *Exoc4* mRNA was quantified by RT-qPCR. As shown in Figure 4C, the *Exoc4* expression level decreased in the kidneys of *Exoc4* knockout mice. Protein degradation in lysosomes is a vital step in renal proximal tubule protein reabsorption. Renal proximal tubule-specific knockout of the *Exoc4* gene resulted in albumin accumulation in the brush border of proximal tubular epithelial cells 14 days after tamoxifen administration (Figure 4D). However, we did not observe the proteinuria phenotype in *AMN* ^*CreERT2*^ *; Exoc4* ^*fl/fl*^ mice 14 days and 60 days after tamoxifen administration (Supplementary Figure S2). Our results suggest that *Exoc4* knockout in renal proximal tubular epithelial cells using *AMN* ^*CreERT2*^ mice could lead to protein degradation defect in the lysosomes of these cells.

## Discussion

In this study, we describe the generation and characterization of a renal proximal tubule-specific *AMN* ^*CreERT2*^ knock-in mouse strain under the control of the endogenous *AMN* gene promoter. The CreERT2-mEGFP was expressed specifically in renal proximal tubules in *AMN* ^*CreERT2*^ mice. When bred with *Rosa26-LSL-tdTomato* reporter mice, highly efficient Cre-mediated recombination occurred, specifically in renal proximal tubular cells, after tamoxifen administration. Compared to *GGT-CreERT2* and *SLC34a1-CreERT2* lines, which express Cre-ERT2 fusion protein in the S3 segment of the proximal tubules and cortical proximal tubules, respectively, *AMN* ^*CreERT2*^ strain expresses Cre-ERT2 in both cortical and medullary proximal tubules, suggesting that it could be used to delete genes in all the renal proximal tubular cells.

As a proof-of-principle study, we showed that renal proximal tubule-specific knockout of *Exoc4* using *AMN* ^*CreERT2*^ led to albumin accumulation in renal proximal tubular epithelial cells. Previous studies showed that tissue-specific knockout of cubilin or megalin in renal proximal tubules does not lead to proteinuria in mice (Amsellem et al., 2010; Nielsen et al., 2016); thus, we chose the *Exoc4* subunit of the exocyst complex to evaluate gene knockout effectiveness using this *AMN* ^*CreERT2*^ mouse line. Our expectation was to investigate the proteinuria phenotype in *Exoc4* knockout mice. Regrettably, we did not observe a substantial rise of proteins in either spot urine or 24-h urine samples obtained from *Exoc4* knockout mice. Protein degradation in lysosomes is one of the major steps in renal proximal tubule protein reabsorption. We examined protein accumulation in renal proximal tubular epithelial cells because we thought that the exocyst complex might affect protein degradation in lysosomes due to the blockage of membrane fusion between late endosomes and lysosomes. Albumin accumulation was observed in renal proximal tubular cells of *Exoc4* knockout mice, suggesting that *Exoc4* knockout in the renal proximal tubular epithelial cells could lead to protein reabsorption defect by blocking protein degradation in the lysosomes.

There are several possible reasons for not observing the proteinuria phenotype in *Exoc4* knockout mice. About 50% of *Exoc4* mRNAs were knocked down after medium-dosage tamoxifen induction in *AMN* ^*CreERT2*^ *; Exoc4* ^*fl/fl*^ mice. The recombination may only occur in 50% of cells in all proximal tubular epithelial cells, resulting in no detectable albumin in urine due to the existence of wild-type cells. When we performed similar experiments using high-dose tamoxifen, mice died quickly after the fourth injection. We speculate that it may take a long time to entirely block the cubilin/AMN/megalin-mediated protein reabsorption process after *Exoc4* is knocked out in renal proximal tubules, leading to proteinuria eventually. We will keep tracking these mice, and hopefully, we can observe the proteinuria phenotype in the future. Nevertheless, our results suggest that this new AMN^CreERT2^ can be used to effectively knock out genes in renal proximal tubular epithelial cells to study the function of these genes in the kidney.

In summary, we have established a novel *AMN* ^*CreERT2*^ knock-in mouse line that expresses Cre-ERT2 fusion protein under the control of the endogenous *AMN* gene promoter in renal proximal tubules. This mouse model will become an invaluable tool for knocking out target genes in proximal tubular cells and studying their function in normal and disease conditions.

## Data Availability

The original contributions presented in the study are included in the article/Supplementary Material; further inquiries can be directed to the corresponding authors.

## Supplementary material

SUPPLEMENTARY FIGURE S1 Co-localization of AMN and megalin in renal proximal tubules. Immunohistochemistry staining of serial sections shows the co-localization of AMN (top) and megalin (bottom). The black and green rectangles depicted in the left column are shown at a higher magnification in the right column, respectively. G denotes glomerulus. Scale bars = 50 μm.

SUPPLEMENTARY FIGURE S2 Knockout of the Exoc4 gene, specifically in renal proximal tubules of AMNCreERT2/+; Exoc4fl/fl mice, did not result in proteinuria. 24-hour urine protein excretion of AMNCreERT2/+; Exoc4fl/fl mice 2 weeks **(A)** or 8 weeks **(B)** after tamoxifen or vehicle administration. No statistical significance was observed (ns). **(C, D)** Representative images of SDS-PAGE gel of spot urine samples from *AMN* ^*CreERT2/+*^ *; Exoc4* ^*fl/fl*^ mice 14 days **(C)** or 60 days **(D)** after tamoxifen or vehicle administration, with equal amounts of mouse urine loaded.
