# Supplementary figures and images for: Generation and characterization of an inducible renal proximal tubule-specific CreERT2 mouse
Source: Front Cell Dev Biol. 2023 May 5;11:1171637. doi: 10.3389/fcell.2023.1171637 (PMC10196630; doi:10.3389/fcell.2023.1171637)

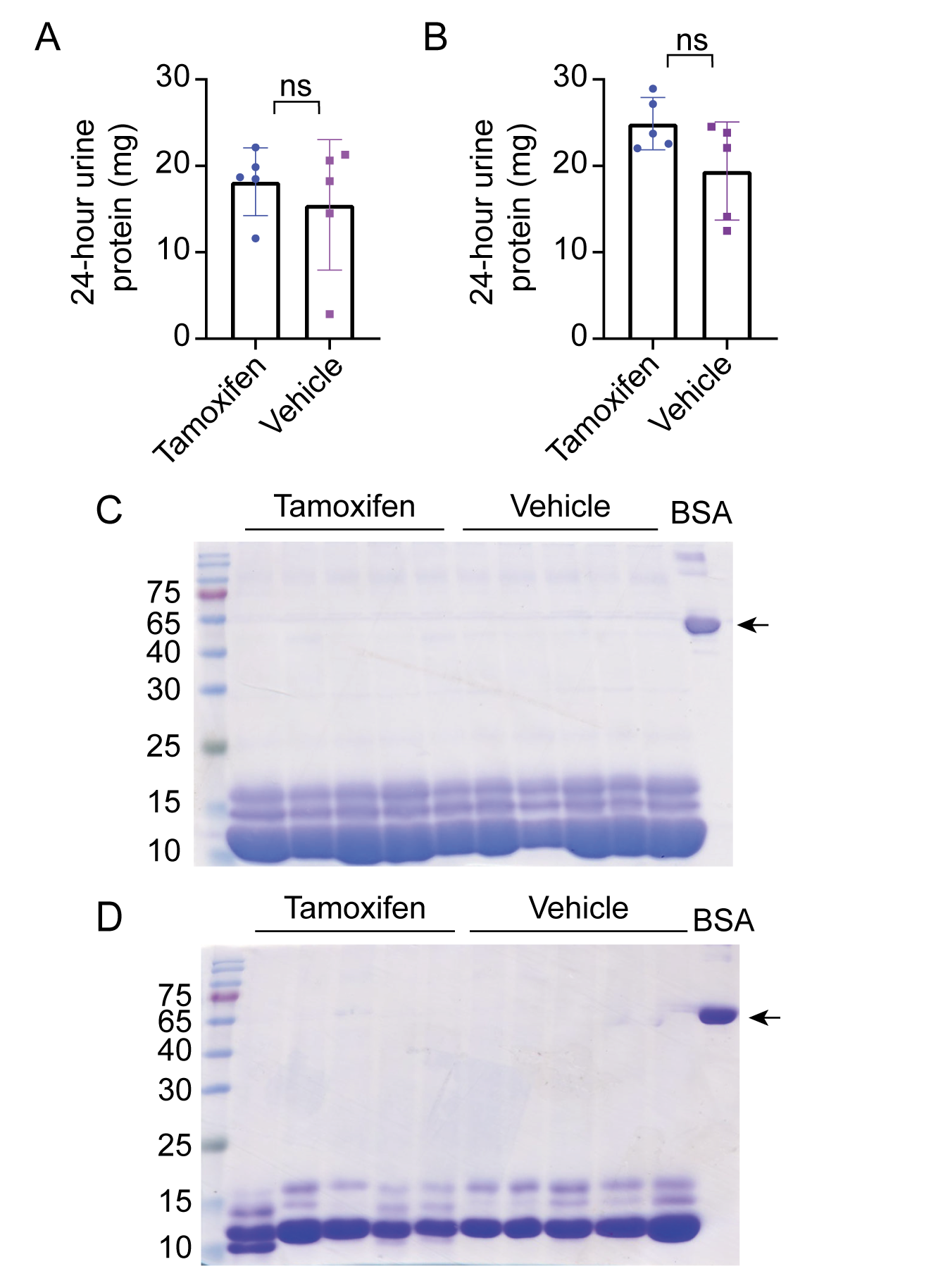

Supplement: Supplementary file 1 [file Image2.TIF]

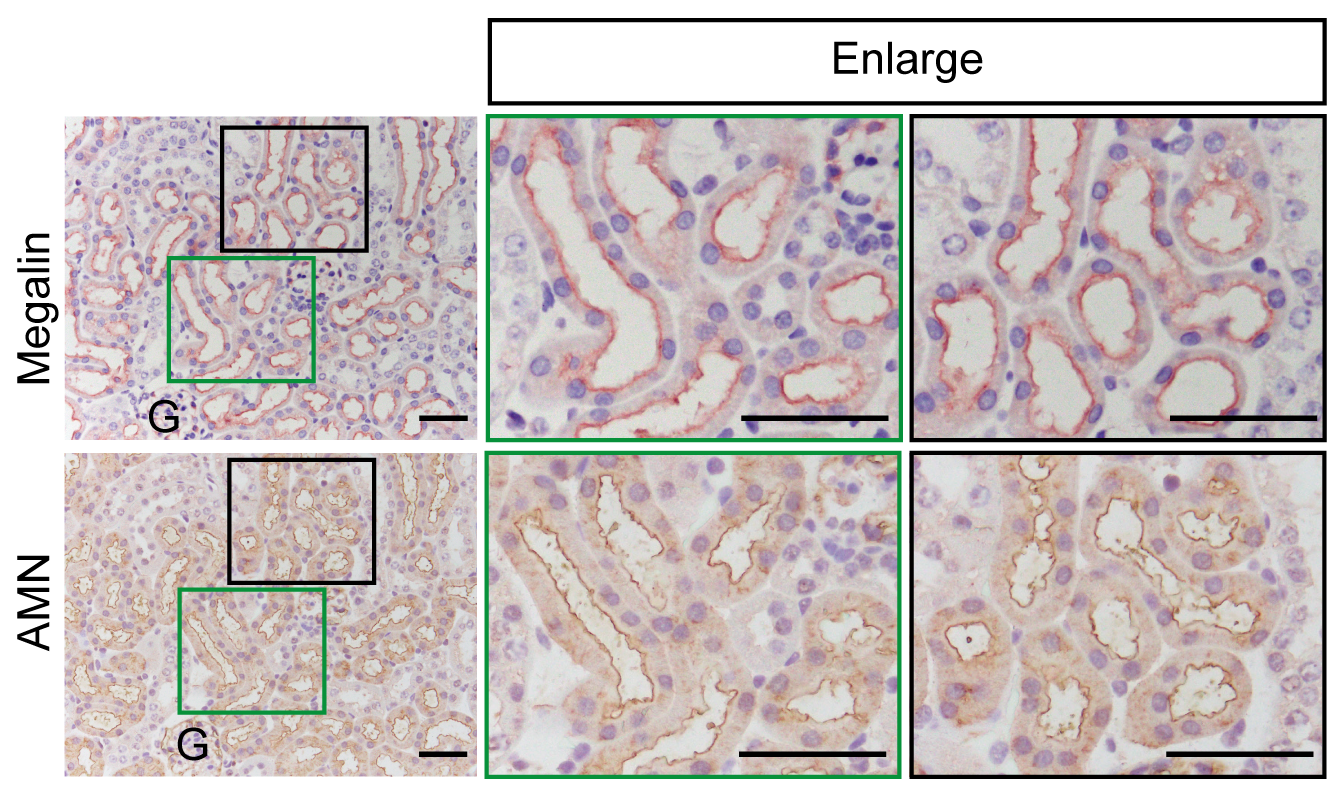

Supplement: Supplementary file 2 [file Image1.TIF]
